# Comparison of the Initial Growth of Different Poplar Clones on Four Sites in Western Slovakia—Preliminary Results

**DOI:** 10.1007/s12155-020-10227-3

**Published:** 2021-01-16

**Authors:** Dávid Heilig, Bálint Heil, Christoph Leibing, Heinz Röhle, Gábor Kovács

**Affiliations:** 1 Institute of Environmental and Earth Sciences, Faculty of Forestry, University of Sopron, P.O. Box 132, Sopron, H-9400 Hungary; 2 Ökoforestino Kft., Ibolya út 11. V/21, Sopron, H-9400 Hungary; 3 IKEA Industry Slovakia, Továrenská 2614/19, SK-901 01 Malacky, Slovakia; 4 Institute of Forest Growth and Forest Computer Sciences, Faculty of Forestry, Geo and Hydro Sciences, Technische Universität Dresden, Pienner Str. 8, D-01737 Tharandt, Germany

**Keywords:** SRF, Hybrid poplar, Growth comparison, Survival, Water availability, Central Europe

## Abstract

This study was conducted to evaluate four hybrid poplar comparison tests along a groundwater availability gradient in Western Slovakia. The weather fluctuation during the 3-year study period was described with indices, such as the Forestry Aridity Index (FAI) or the hydrothermal coefficient (HTC). The soil chemical and physical parameters were determined from soil samples from the two upper horizons. The nutrient status and supply of the trees were categorized based on leaf elemental analysis. Altogether, 21 different clones from 6 genomic groups were compared. The survival (SRV), diameter at breast height (DBH), and height of the trees (H) had been measured annually since the plantations were established, and from these measurements, mean annual height increment (MAHI) values were derived. These weather, edaphic, and clonal factors were evaluated and compared. Significant effects of the site (edaphic factors) were found as the primary source of variance and clonal differences as secondary sources of variance among the growth of trees. The interaction of site × clone effects was not significant. The results showed that for short rotation forestry (SRF), the site parameters—especially groundwater availability—are key factors.

## Introduction

The demand for softwood has grown substantially in recent years, while climate change started to threaten the future supply [1]. Warm winters and bark beetle damage accompanied with storms and droughts are endangering forests all over Europe [2]. The coronavirus pandemic also works against the global wood market. To be prepared for a future wood shortage, one possible effective risk management strategy is the local resource production in plantations. This requires new policies and strategic decisions on the choice of tree species supported by quantified information on extreme weather events (e.g., storms and drought) exacerbated by climate change.

Since the oil crisis in the 1970s, the establishment of short rotation plantations (SRP) started to become popular, at first in the Scandinavian region [3]. At the millennium, a new growing interest started to rise in Europe for SRP, too [4]. The plantations were considered a renewable source of biomass [5] and an environmental service [6]. SRP established with *Populus* species and their hybrids have already been an integral component of environmental sustainability portfolios worldwide [7]. The biomass can be used for several purposes, such as the production of lightweight boards, bioenergy, biofuels, and bioproducts. The risks, opportunities, and the history of poplar cultivation in the Central European region [4] brought the Dendromass4Europe project (D4EU) to life.

D4EU aims to start SRP on marginal agricultural lands, especially in Western Slovakia, to secure a biomass supply for lightweight particle board production. Along this process, several beneficial effects are expected, such as the creation of job opportunities in rural areas [8]. This allows realizing substantial savings of material and costs in the production of particle boards, relieving pressure from natural forests wood supply of the timber industry, and it even has a high potential for carbon storage [9] or controlling soil erosion [10]. The first results of the D4EU project [11] confirmed earlier findings [12, 13] that newly established SRP provide improved habitat and increased plant diversity on former agricultural farmlands. Gaps and small edge-like habitats can further improve the overall value of SRP in terms of biodiversity.

The success of SRP is also measured in the amount of produced biomass. The growth of the trees and therefore the yield of the plantations depend on several components, such as edaphic factors, weather, clonal differences, and plantation management [14, 15]. The edaphic factors are the soil’s hydrological state [16] and the soil itself both in terms of physical and chemical parameters or nutrient supply of the soil [17]. Tužinský [18] showed that sandy soils with high drainage, accompanied by a dense root system, can prevent high saturation of soils during the growing season. This emphasizes the importance of groundwater as an available source of water for plants which can reach at least the capillary fringe during dry periods. Flooded soils have reduced growth potential as well, due to their anaerobic conditions [19] and to the phytotoxins which are by-products of the reductive processes [20]. Therefore, the effects of groundwater availability for plants can be beneficiary but also limit the root growth.

The poplar genus is known for its vigor, fast growth, and adaptability to rapid environmental changes [21, 22]. The aim of the study was to identify well-adapted hybrids to a given range of environmental and edaphic conditions. This results in deviations of the growth performance of the groups and even the members of the groups on the given sites. There are differences between the growth strategies of different hybrids and their reactions to different environmental factors [23] generally summarized under the term genotype by environment (G × E) interaction.

In this study, we investigated the first 3-year performance of different poplar clones along a groundwater availability gradient. The main objectives of this paper are (1) to identify the environmental factors which have significant influence on the growth of the different hybrids, (2) to show how different hybrid poplar clones perform in Western Slovakia in short rotation plantations, and (3) to select which clones have the highest growth along an environmental gradient.

## Materials and Methods

### Site Locations and Description

**Fig. 1:**
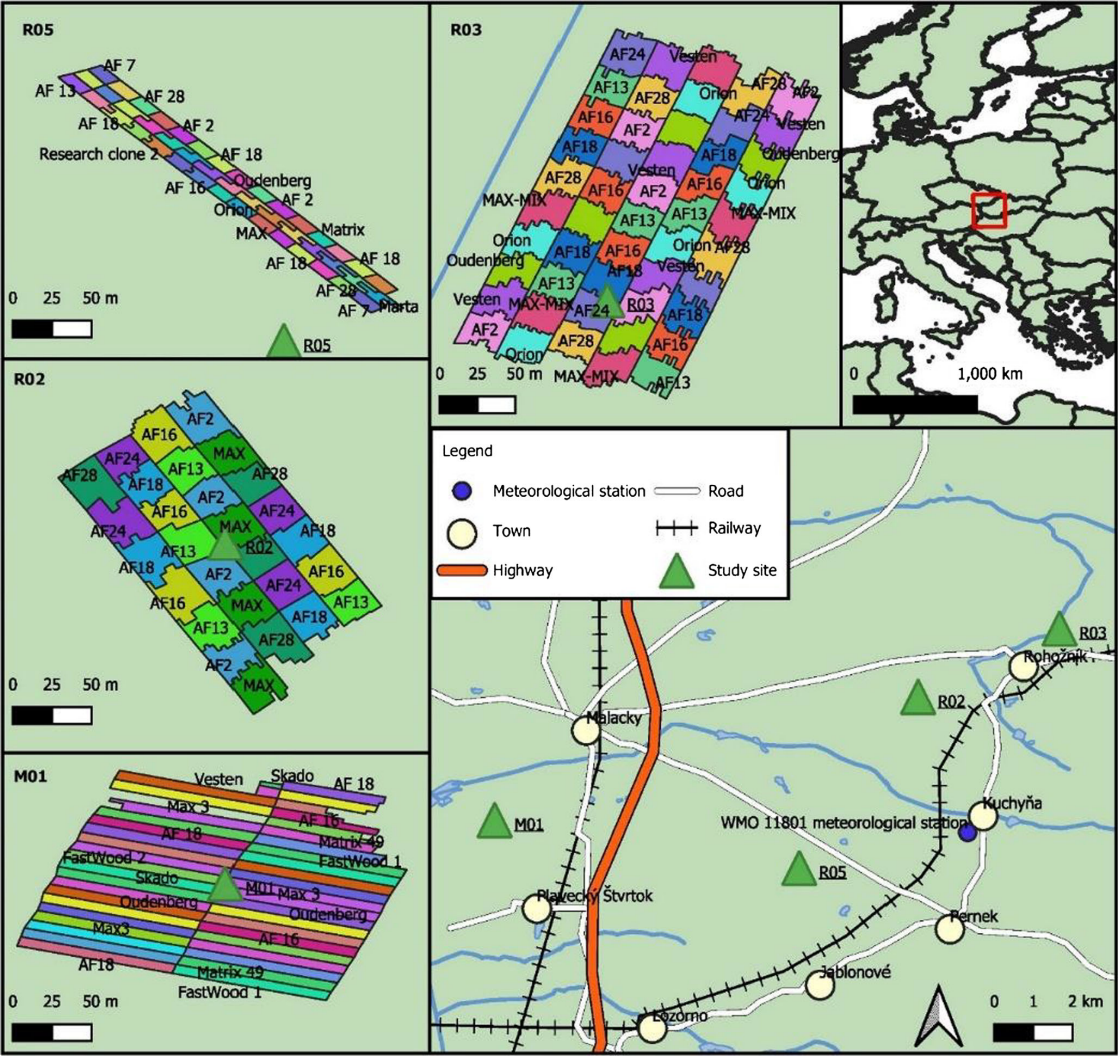
Location of the experimental sites and layout of the study areas

The poplar SRP are located in Záhorská lowland, Slovakia (Fig. 1). According to the Köppen-Geiger classification, warm temperate, fully humid, and hot summer climate (*Cfb*) characterize the region [24]. Long, warm, and dry summers and mild, short, and very dry winters and short transitional periods are typical [25]. Table 1 shows the annual and growing season temperature means and sums of precipitation, measured at Malacky (WMO-11801) weather station from 2014 till 2018 [26]. The length of the growing season is the number of days when the daily mean temperature is above 10 °C. Based on these data, the Forestry Aridity Index (FAI) [27] was determined for the time period of the study (2014–2018). Increasing FAI values indicate drier years. These values are divided into categories which represent forest types. FAI values show beech climate under 4.75, hornbeam-oak climate between 4.75 and 6.00, sessile oak-turkey oak climate between 6.00 and 7.25, and forest-steppe climate above 7.25. The weather highly fluctuated in the study period. On average over the 5 years, the annual temperature*** and growing season temperature** and growing season lengths* were slightly higher than the averages determined by former studies for the period of 1997–2002 [28] and 1901–1995 [18], while annual precipitation and precipitation in the growing season showed no significant difference (* is significant at α = 0.05, ** is significant at α = 0.01, and *** is significant at α =0.001). The hydrothermal coefficient was calculated based on Selyaninov’s formula [29]. Three categories were used to classify the results [30]: sufficiently humid above 1.0, moderate arid conditions between 1.0 and 0.7, and coefficients under 0.7 were classified as very arid.

**Table 1:** Weather of Malacky, Slovakia, during the study period (2014–2018)

Year	Annual		Growing season			FAI^a^	HTC^b^
	Mean temperature [C°]	Precipitation [mm yr^-1^]	Length (base 10 °C) [days]	Mean temperature [C°]	Precipitation [mm yr^-1^]		
2014	11.3	759	215	16.0	604	4.97	1.75
2015	11.3	500	179	18.4	282	10.02	0.86
2016	10.6	596	186	17.3	350	6.29	1.09
2017	10.5	499	208	16.7	310	11.15	0.89
2018	11.8	743	215	18.3	585	4.12	1.49
Mean	11.1	619	201	17.3	426	7.31	1.22
SE	0.2	57	8	0.4	69	1.39	0.17

^a^Forestry Aridity Index [22]

^b^Hydrothermal coefficient [25]

The four sample plantations are located in the Slovak cadasters of Rohožník (R02 and R03), Pernek (R05), and Plavecký Štvrtok (M01). The first two are on the central plateau of the lowland, characterized by smooth scattered mounds, and depressions filled with wind-blown sands. M01 is located on the western part of the lowland, which was formed by fluvial activities and was partly covered by sand as a result of aeolian processes [31]. The differences in the reliefs are also shown by elevations above sea level depicted in Table 2.

**Table 2:** Site name, locations, establishment years, groundwater levels, and soils of four test plantations in Slovakia

Site name	North latitude	East longitude	Elevation [m]	Establishment year	Groundwater level [cm]	WRB soil class^a^	Soil texture
R05	48.388567°	17.091752°	205	2016	> 200	Arenosol	(coarse) sand
R02	48.446178°	17.132799°	200	2016	160	Gleysol	sandy loam
M01	48.403759°	16.987345°	155	2017	120	Gleysol	sand
R03	48.470154°	17.181804°	190	2016	< 60	Gleysol	sandy loam

^a^World Reference Base (WRB) [32]

On the sandy parent materials, under hydromorphic conditions, the processes formed Gleysols, except in plantation R05, where the soil was not affected by groundwater. Surplus water can be found only after wet periods. The other plantations can access groundwater, but at different scales (Table 2). The dominant soil texture is sand, but the proportion of the fine materials is different. It has a significant effect on the water storage capacity of the soils.

### Laboratory Analyses

Soil auger profiles were obtained in representative points of the plantations (R02, R03, and R05 in 2015 and M01 in 2017). At the same time, groundwater levels were measured, but the exact fluctuation of the levels is unknown. The samples were collected from the upper two horizons. General soil chemical and physical analyses were performed, such as pH determination with water as a suspension medium [33], organic carbon content determination with the volumetric method [34], soil texture analysis [33], and total CaCO_3_ content measurement with the Scheibler apparatus. The nitrate in the soil samples was measured by the phenoldisulphonic acid method [33]. The ammonium lactate (AL) soluble phosphorus content is based on UV/VIS spectrophotometry [35] and the AL soluble potassium content was determined by atomic absorption spectrophotometry [35]. Magnesium content was determined by the acid digest with EDTA titration method [33]. Sulphate content measurement was extracted by 1 M KCl solution [36]. The analyses were performed by an accredited soil laboratory (Tanakajdi Talajvédelmi Laboratórium). The results of the soil analyses are shown in Table 3. No samples were collected from R05. The results are evaluated based on the soil nutrient supply categories which were set up by Buzás [36], originally for agricultural use. The limits are for sandy soil texture. The soil is well-supplied with AL-P_2_O_5_ above 100 mg kg^-1^ and poor under 60 mg kg^-1^. The plant available potassium content under 81 mg kg^-1^ is low and medium between 81 and 120 mg kg^-1^. The Mg content is in the medium category between 40 and 60 mg kg^-1^. Terelak [37] set up categories for sulfate sulfur (SO_4_^2-^-S) content of mineral soils. Top horizon samples with SO_4_^2-^-S levels between 0.1 and 500.0 mg kg^-1^ are poorly supplied.

**Table 3:** Soil chemical parameters and nutrient contents of the two upper soil horizons in study sites

Site name	Layer [cm]	pH (H_2_O)	CaCO_3_ [%]	Organic C [%]	NO_3_^-^ + NO_2_^-^ - N [mg kg^-1^]	AL-P_2_O_5_ [mg kg^-1^]	AL- K_2_O [mg kg^-1^]	Mg [mg kg^-1^]	SO_4_^2- ^- S [mg kg^-1^]
R02	0–30	6.4	< 0.1	1.8	14.2	136.0	80.0	22.4	4.4
	30–70	7.4	0.2	0.5	9.3	32.0	121.0	62.5	3.1
M01	0–40	7.8	0.5	0.8	7.1	334.0	104.0	65.3	2.4
	40–90	8.1	2.0	0.3	24.8	38.0	51.0	56.2	3.2
R03	0–30	7.8	1.8	0.8	3.2	401.0	112.0	37.4	1.5
	30–80	8.1	4.0	0.5	2.6	210.0	66.0	61.1	2.3

Leaf samples were collected at R02, R03, and M01 sites in the second half of August 2018. Three adjacent trees were sampled. About 8–10 healthy and mature leaves were collected from the middle section of sylleptic branches closest to the apical bud. The samples were dried at 60 °C, finely ground, and then homogenized. The leaf samples were stored in paper bags in a dark place. The measurements were performed by the Institute of Soil Science and Site Ecology, TU Dresden (TUD-ISSE). The methods are based on the Handbook of Forestry Analysis [38]. The C and N content of the leaves was measured with a CN elemental analyzer. The samples were prepared with the acid pressure digestion method. Leaf Al, B, Ca, Fe, K, Cu, Mg, Mn, Na, P, S, and Zn elements were quantified by ICP measurements. The average and standard error of the samples were calculated on the plantation level. The results were compared with the limits set up by Ulrich [39] and Lyr [40] (Table 4).

**Table 4:** Total leaf nutrient contents on the study sites in 2018 (Means ± Standard Error) and the nutrient content ranges set up by Ulrich [38] and Lyr [39]

Site name	Clone	C [%]	N [%]	P [mg kg^-1^]	K [mg kg^-1^]	
R02	AF2	45.21 ± 0.36	2.93 ± 0.08	4.97 ± 0.31	12.85 ± 3.87	
M01	AF18	46.11 ± 0.49	2.74 ± 0.09	2.70 ± 0.59	21.88 ± 3.73	
R03	AF2	44.04 ± 0.16	2.77 ± 0.06	6.63 ± 0.30	24.80 ± 1.05	
Ulrich limits				1.8–3.0	12.0–15.0	
Lyr limits			2.27-2.77	2.2-2.3	7.5–11.1	
Site name	Clone	Mg [mg kg^-1^]	S [g kg^-1^]	Fe [mg kg^-1^]	Ca [g kg^-1^]	B [mg kg^-1^]
R02	AF2	4.91 ± 0.53	5.70 ± 0.20	56.98 ± 10.77	16.77 ± 0.88	33.69 ± 1.92
M01	AF18	3.11 ± 0.49	4.01 ± 0.20	68.62 ± 4.10	11.30 ± 1.04	55.99 ± 2.46
R03	AF2	3.24 ± 0.16	5.37 ± 0.16	62.09 ± 11.03	18.00 ± 1.44	27.31 ± 0.96
Ulrich limits		2.0-3.0			3.0–15.0	15–40
Lyr limits		2.4-3.5		1310	9.8–10.6	
Site name	Clone	Cu [mg kg^-1^]	Mn [mg kg^-1^]	Zn [mg kg^-1^]	Na [mg kg^-1^]	Al [mg kg^-1^]
R02	AF2	0.07 ± 0.04	21.59 ± 1.80	62.86 ± 9.34	33.09 ± 2.72	8.39 ± 3.93
M01	AF18	0.15 ± 0.06	43.02 ± 3.35	39.85 ± 10.47	83.08 ± 3.58	27.05 ± 1.04
R03	AF2	0.00 ± 0.02	10.49 ± 0.53	91.73 ± 9.75	12.62 ± 7.45	23.41 ± 10.65
Ulrich limits		6–12	35–150	15–50		
Lyr limits			250–350			

### Plantation Establishment and Maintenance

Plantations were established in 2016 (R02, R03, and R05) and 2017 (M01) with a similar planting protocol on every field. R02 and R05 were established on meadows, while M01 and R03 sites were previously used as farmland. The machinery, personnel, and quality of planting material were different, which introduces some uncontrolled variability in the experiment. The study plantations originally were established for comparing different hybrids to help choosing the clone with the best growth on the site. Several different poplar hybrids were planted. The planting was done into prepared soil and with the slit method. The planting depth was 60 cm in general. Long rods of 110–170 cm without apical buds were used as planting material. The upper diameters of the rods were not recorded for every individual one, but it ranged from 1.5 to 3.0 cm. M01 was planted with 35 cm long cuttings, which were pushed into the soil. The planting stocks were obtained from various sources. Each plantation was planted in early spring. Altogether 21 different hybrids (Table 5) were used in the tests to compare their initial growth and in the future yields. The hybrids were divided into 6 genomic groups along their parentage. In this study the two most numerous groups are *Populus deltoides × P. nigra* (DN) clones, *P. trichocarpa × P. maximowiczii*, and *P. maximowiczii × P. trichocarpa* clones (TM). The group of *P. deltoides × P. deltoides* (DD) consists of two research clones. The other groups have only one clone, or a clone mixture, *P. nigra × P. maximowiczii* (NM), *(P. deltoides × P. nigra) × (P. deltoides × P. trichocarpa)* (DNDT), and *P. alba* (A) (Table 5.). The tests follow a random block pattern. The shape of the plantation also affected the network of blocks and the number of trees in blocks. The number of trees in a row varies between 15 and 50 trees. The statistical minimum requirement was to have at least 15 measurable trees in a block and at least 3 repetitions in separate blocks within a plantation.

**Table 5:** List of the planted clones along with their parentage and genomic group, and the number of measured blocks and trees in the study sites

Clone	Parentage	Genomic group	Number of blocks/trees in blocks			
			R05	R02	M01	R03
*Populus* ‘Marte’	*P. alba*	A	3/20			
*Populus* ‘AF2’	*P. deltoides × P. nigra*	DN	3/20	5/15	3/50	5/30
*Populus* ‘AF7’	*(P. deltoides × P. nigra) x (P. deltoides x P. trichocarpa)*	DNDT	3/20			
*Populus* ‘AF13’	*P. deltoides × P. nigra*	DN	3/20	4/15		5/30
*Populus* ‘AF16’	*P. deltoides × P. nigra*	DN	3/20	4/15	3/50	5/30
*Populus* ‘AF18’	*P. deltoides × P. nigra*	DN	6/20	4/15	3/50	5/30
*Populus* ‘AF24’	*P. deltoides × P. nigra*	DN		5/15		5/30
*Populus* ‘AF28’	*P. deltoides × P. nigra*	DN	3/20	5/15		5/30
*Populus* ‘Orion’	*P. deltoides × P. nigra*	DN	3/20			5/30
*Populus* ‘Oudenberg’	*P. deltoides × P. nigra*	DN	3/20		2/50	5/30
*Populus* ‘Vesten’	*P. deltoides × P. nigra*	DN	3/20		3/50	5/30
Research clone 1	*P. deltoides × P. deltoides*	DD	3/20			
Research clone 2	*P. deltoides × P. deltoides*	DD	3/20			
*Populus* ‘Bakan’	*P. trichocarpa × P. maximowiczii*	TM			3/50	
*Populus* ‘Skado’	*P. trichocarpa × P. maximowiczii*	TM			3/50	
*Populus* ‘Max’ mixture	*P. maximowiczii × P. nigra*	MN	3/20	5/15	3/50	5/30
*Populus* ‘FastWOOD 1’	*P. maximowiczii × P. trichocarpa*	TM			3/50	
*Populus* ‘FastWOOD 2’	*P. maximowiczii × P. trichocarpa*	TM			2/50	
*Populus* ‘Hybride 275’	*P. maximowiczii × P. trichocarpa*	TM			2/50	
*Populus* ‘Matrix11’	*P. maximowiczii × P. trichocarpa*	TM			2/50	
*Populus* ‘Matrix49’	*P. maximowiczii × P. trichocarpa*	TM	3/20		3/50	

The planting grid was 3 x 2 m (1 667 tree ha^-1^) which makes mechanical weed control possible between rows. The disk harrows were used twice, but at M01 three times, in the first year and once during the subsequent years. This procedure reduces the effect of weed competitors. During the study period neither fertilization, nor irrigation, nor herbicide was applied, but prior to planting of M01, a pre-emergent herbicide was used. Chemicals were not used at the other sites. After the first growing season, the trees were pruned at every plantation. The aim of this procedure is to achieve straighter and more cylindrical stems and to eliminate the trees with multiple stems. The plantations are planned to be harvested on a 5-year basis, for four rotations. Plantation R02 and R03 were fenced against game browsing. A fence was erected around R05 only after the first growing season (after heavy browsing damage). M01 was not protected against game damage during the study period.

### Growth Measurements

Since the plantations have not yet reached harvest age, only non-destructive measurements were done in their dormancy period (November–April). The circumference at breast height (CBH) (measured at 1.3 m height) and height (H) was measured annually at individual tree levels. The diameter at breast height (DBH) was calculated from the CBH. For this we assumed the cross-section of the trees as a circle. All measurements were performed on inner rows of each block. The CBH of every tree was measured with a tape measure. The measurements were done with mm accuracy. The heights of trees were measured at dm accuracy. Two different devices were used for height measurements. Under 10-m height, a telescopic scaled rod was used and above that a digital trigonometric height measurement device combined with a laser distance measuring device.

In the 1-year old plantations, carrying out of CBH measurement is often problematic. A small circumference can lead to measurement errors. Another issue is that if the initial height growth is weak and the trees do not reach 1.3 m height consequently, they have no CBH. Therefore, in the first year, only H was measured, which describes the blocks better, but in the subsequent year, both CBH and H were measured.

The survival was based on measurements done in the dormancy period. The trees with damaged buds or dried dominant stems often have intact root systems which could result in regrowth of the stem. The trees which did not show any signs of life were recorded, but they were not measured. The mean survival (SRV) was calculated as a proportion of the number of measured and planted trees and expressed as a percentage value. Quadratic-mean diameter was calculated as the mean DBH. Mean H was based on Lorey’s mean height [41] calculation method, where individual trees were weighted in the proportion of basal area at breast height. Height increment is calculated on a single tree level as a difference of H in consecutive years. The first-year increment is equal to the H for cuttings, and it is the difference between the above ground part of the rod (50–110 cm) and H for the plantations established with rods. Arithmetic means of the H are calculated on block level for every year, and the mean annual height increment (MAHI) is calculated as the arithmetic mean of these values.

The signs of game browsing were not recorded on an individual basis, but on a block basis in the form of a general description. There were two typical types of damage. The first when the dominant stem was broken and/or the apical bud was damaged and the second was when the bark was rubbed. Both resulted in worse stem shape, lower survival, and growth rate.

### Statistical Data Analyses

The data were organized into MS Excel spreadsheets, where the means, standard deviations (SD), and standard error of means (SE) were calculated. Means and SE are represented on the figures. The statistical and analytical methods suggested by Petersen [42] and Gotelli [43] were used. T-test were used to compare weather data with the climate data of former studies [18, 28]. Pearson correlations among weather parameters and MAHI of the four sites along the environmental gradient were calculated. A one-way analysis of variance (ANOVA) was performed for every site at the level of blocks with at least 67% SRV; to compare on-site differences of hybrids in terms of SRV, DBH and H were measured in 2018. The means were divided into homogenous subsets with the Tukey HSD method. MAHI was calculated for genomic groups. A two-way ANOVA was conducted at block level means of survival and annual height increment. The calculations were done in IBM SPSS Statistics for Windows, version 26.0. The differences were tested at levels of significance of 0.001, 0.01, and 0.05. Tukey’s HSD tests were performed to compare the means (*α* = 0.05). These procedures were performed only on the five most common hybrids, which had at least a 67 % survival rate (omitting blocks with enlarged growing space and providing enough repetition of statistical analyses) and were planted on at least three sites.

## Results and Discussion

### Environmental Factors as Sources of Site Effect

The sites are affected by several different parameters, such as weather, groundwater availability, and soil fertility. The weather varied greatly during the investigated years (Table 1). The weather factors showed no statistically significant correlations with the MAHI as the result of the short observation period (3 years), but future results may be worth exploring. Wang and McFarlane [44] and Miller [14] described a close connection between weather indices, like growing degree days or annual precipitation and MAHI. In general, the cold and especially the dry periods showed lower growth rates than warm periods with sufficient precipitation. The performance of ‘AF2’ based on a Latvian study [45] was lower than the ones we measured in Slovakia, probably because of the shorter and colder Latvian growing seasons. Šēnhofa et al. [32] showed the intra-year height increments of the hybrid poplars depend on the precipitation. Consequently, the most intensive growth is during June–July in Latvia.

The chemical characteristics of the soil are shown in Table 3. The pH values were close to neutral, but there were slightly acidic and alkalic horizons. The soil lime levels were in the optimal range, except in the case of R02. That soil profile was low in calcium-carbonate, accompanied by a slightly acidic pH levels due to leaching and non-calcareous parent materials. The organic carbon (OC) content of the soil in R02 was high, as this site had been a pasture before the establishment of the plantation, while the other two sites (R03 and M01) had been croplands where, as a result of the intensive agricultural land use, the OC content supply is lower [46]. According to the levels determined by Buzás [36], the macronutrients were at low-medium levels on all the sites. The plant-available nitrogen contents (NO_3_^-^ + NO_2_^-^ - N) are generally low, but differences can be found among the sites. Total N content—derived from OC content—differs from the measurements, but it also shows low N amount in the soils. Phosphorus content available to plants is high in every top horizon and in lower horizon at R03, while in the other lower horizons, it is low. AL- K_2_O content is in the poor and middle levels. Magnesium in the soils is around the limit between medium and low (60 mg kg^-1^). The sulfate content is in the low range, but it is sufficient for agricultural use [36, 37].

The results of leaf nutrient analyses are presented in Table 4. The overall leaf nutrient status was good, and the differences among the fields are relatively low. Compared with the limits set up by Lyr [39] and Ulrich [38], most of the macronutrients (C, P, Ca, Mg, S) were present in a higher amount, while N and K was consistent with the values in the mentioned papers. The micronutrient status of the leaves was similarly good. The Zn content of the leaves was higher than the optimal range, but there were no visible signs of phytotoxicity, and according to Chaney [47], above soil pH 5.0–5.5, the occurrence of Zn phytotoxicity is low. Cu, Na B, and Al levels were within the optimal range. The Fe and Mn levels were low compared with the ranges of Lyr [39], but a more recent study [48] focusing especially on iron deficiency of hybrid poplars found well-supplied Fe levels similar to our measurements. In R05 neither soil analysis nor leaf nutrient analysis was performed. The soil belongs to the type of Arenosol, which is poor in nutrients in general [49]. Altogether the soil nutrient levels were lower than the optimal levels for agricultural use, and the poplar leaves did not show any nutrient deficiency. Instead, in some cases, nutrient abundance occurred similarly to an Estonian study [50], which showed no significant changes in macronutrient levels of the soil after the first rotation of hybrid aspen.

The sites were organized along a hydrological gradient (Table 2), where R05 has only the precipitation as water source. R02 has access only to the groundwater periodically during the intensive growing phase in spring to early summer; M01 can tap into groundwater throughout the whole year, as well as R03, but here the high groundwater level can limit the growth and, in some cases, can threaten the survival of trees. Fan et al. [51] described interactions of groundwater and rooting depth. In R03, the near surface groundwater level (< 0.60 m) reduced the root growth and the overall tree growth due to oxygen stress. In wet years, the possible rooting depth can be even more reduced, while in dry years the sinking groundwater level allows more space for root growing.

Our results have led us to the assumption that the survival and growth variation of the fields are highly based on water availability, similar to the finding of Schmidt et al. [52].

### Performance of Hybrid Poplar Clones on Different Sites

**Fig. 2:**
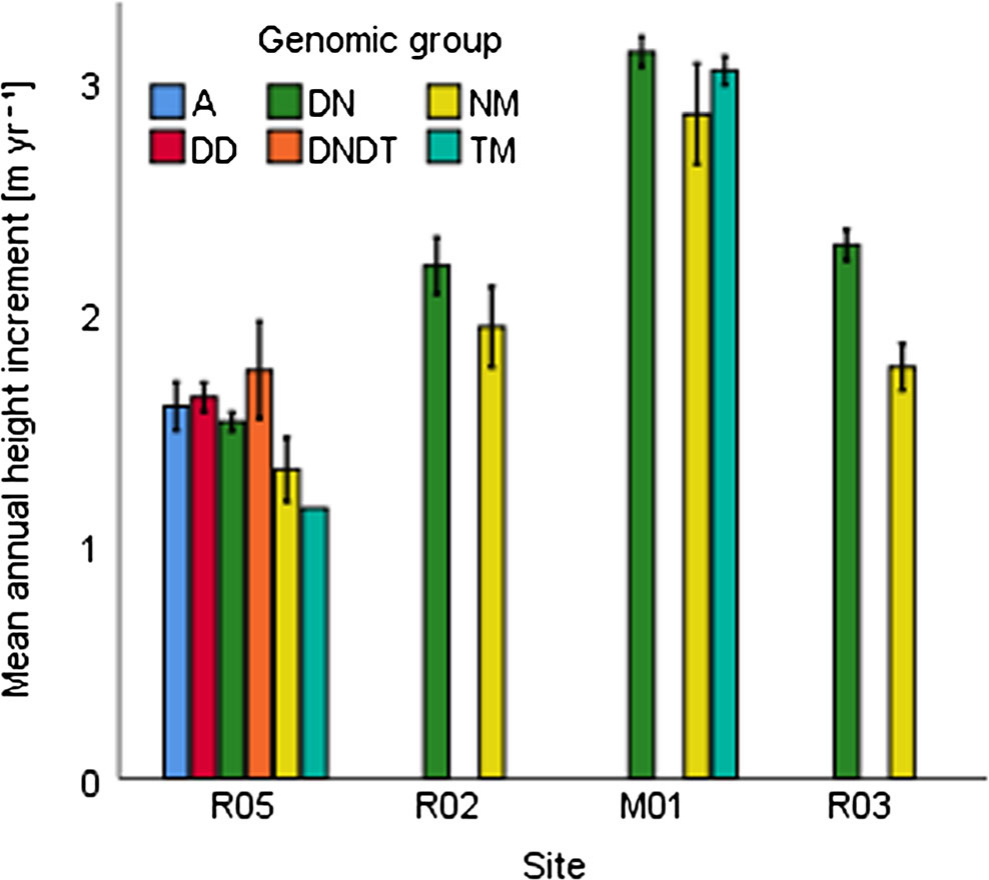
Mean annual height increment and standard error of genomic groups along the study sites. In the case of M01 mean is calculated based on 2 growing seasons, while in the other cases it is based on 3 seasons

The growth deviations were not only the results of differences in their edaphic character but also the effect of the differences of genotypes. This makes the proper clone selection an essential part of the optimization of growth, beside the site selection. In our study the most efficient genomic group was DN in general. These clones tolerated dry periods well and thrived under moist conditions. The MAHI was between 1.5 and 3.1 m (Fig. 2). The clones in this group reacted differently to site factors. ‘AF13’ and ‘Orion’ had relatively low survival rates and growth without access to groundwater, while both were among the best under wet conditions. ‘AF24’ and ‘AF28’ showed low growth and survival rates along the groundwater accessibility gradient. ‘AF2’, ‘AF16’, ‘AF18’, ‘Oudenberg’,  and ‘Vesten’ were generalists in the sense of survival, and they showed increasing growth along the improving conditions (Table 6). It must be noted that the low survival of ‘AF2’ at sites R03 (Table 6) was due to unknown factors. In the buffer zone around the test area, there was also ‘AF2’, but no signs of low survival were observed.

**Table 6:** Mean survival (SRV), diameter at breast height (DBH), and height (H) of the clones along the study sites, measured after growing season of 2018, plantation age is presented in the brackets

Clone	R05 (3 yr)			R02 (3 yr)			M01 (2 yr)			R03 (3 yr)		
	SRV [%]	DBH [cm]	H [m]	SRV [%]	DBH [cm]	H [m]	SRV [%]	DBH [cm]	H [m]	SRV [%)]	DBH [cm]	H [m]
Research clone 1	75^a^	4.8^ab^	5.5^a^									
Research clone 2	92^a^	6.8^b^	6.2^a^									
'AF2'	92^a^	6.0^ab^	6.0^a^	82^a^	8.6^a^	8.7^a^	43	7.5	6.4	60	6.8	8.0
'AF7'	98^a^	6.3^ab^	6.5^a^									
'AF13'	38	4.2	5.2	77^a^	7.6^a^	8.7^a^				89^ab^	5.8^a^	7.8^abc^
'AF16'	95^a^	5.0^ab^	6.0^a^	78^a^	6.5^a^	8.6	79^a^	6.7^a^	5.9^a^	89^b^	6.2^a^	8.3^bc^
'AF18'	85^a^	5.2^ab^	5.8^a^	79^a^	7.1^a^	8.4^a^	95^a^	7.3^a^	6.3^a^	83^a^	5.2^a^	7.0^ab^
'AF24'				57^a^	7.4^a^	9.2^a^				13	4.9	6.3
'AF28'	33	4.7	5.4	35	4.3	6.1				0	-	-
'Bakan'							68^a^	6.5^a^	6.2^a^			
'FastWOOD 1'							83^a^	5.9^a^	6.1^a^			
'FastWOOD 2'							79	6.1	6.3			
'Hybride 275'							60	3.9	5.9			
'Marte'	28	4.6	5.6									
'Matrix11'							76^a^	6.1^a^	6.5^a^			
'Matrix49'	27	3.4	4.4				75^a^	5.1^a^	6.4^a^			
'Max' - mixture	62^a^	4.2^a^	5.3^a^	7 5^a^	5.6^a^	7.1^a^	69^a^	4.9^a^	6.1^a^	97^b^	4.8^a^	6.4^a^
'Orion'	47	3.9	5.1							97^b^	6.9^a^	9.4^c^
'Oudenberg'	90^a^	4.5^ab^	5.3^a^				82^a^	5.7^a^	6.5^a^	96^b^	5.8^a^	8.3^abc^
'Skado'							79^a^	5.7^a^	6.0^a^			
'Vesten'	97^a^	5.5	5.8^a^				86^a^	7.3^a^	6.7^a^	98^b^	7.0^a^	8.3^bc^
Mean	69	4.9	5.6	69	6.7	8.1	75	6.1	6.3	72	5.9	7.8
SE	8	0.3	0.1	6	0.5	0.4	4	0.3	0.1	12	0.3	0.3
p<	NS	.05	NS	NS	NS	NS	NS	NS	NS	.01	.05	.01

^abc^ represents homogeneous subsets of means on the level of blocks separately for every site in the case of SRV, DBH, and H. Values without signs were not used for the analyses due to their low survival and/or number of repetitions

SE represents standard errors

NS represents no statistical significance at α = .05

At R05 site, the DBH values of the clones were significantly different (F (8, 17) = 2.832, *p* < .05). The ‘Max’ mixture had a significantly lower DBH value than Research clone 2. The other clones were divided into both groups. R02 and M01 showed no significant differences among the clones—with at least 67% survival. At R03 there was a significant difference in survival (F (8, 17) = 1.836, *p* = 0.14). ‘AF18’ 83% survival is lower than the other clones at R03. Also, DBH showed a significant difference among the hybrids (F (6, 27) = 2.573, *p* < .05), but the only one homogeneous subset was made. The highest difference was observed between ‘Vesten’ and ‘Max’ mixture, and the lowest difference was found between ‘Orion’ and ‘Max’ mixture and ‘AF18’ and ‘Vesten’. H showed significant differences of means (F (6, 27) = 4.223, p < .01), too. Three groups were formed from the results. The ‘Max’ mixture belongs only to the group with the smallest height. ‘Orion’ is the only member of the highest group. According to the Tukey’s HSD test, the rest of the clones are grouped into a middle group, but there are overlaps between the homogeneous subsets.

Studies from colder climate regions suggest the separation of generalist and specialist hybrids [13, 18, 53]. While in colder and probably more humid regions, clones with *P. maximowiczii* parentage are generalists due to their cold tolerance, here the TM and NM groups were specialists for moist conditions. In our case, the SRV of these clones was lower than that of the other groups. This can be the result of a higher sensitivity to dry periods [8, 54], which may often occur during spring in the study region. Late or early frosts can threaten these clones, too [46]. The NM group consists of a mixture of ‘Max1’, ‘Max3’, and ‘Max 4’ in an equal proportion, referred to as ‘Max’ mixture in this paper. This mixture has a generally low growth rate, and its survival was better on sites with high groundwater levels. TM clones ‘FastWOOD 1’, ‘FastWOOD 2’, ‘Hybride 275’, ‘Bakan’, ‘Skado’, and ‘Matrix 11’ were only planted on M01, but the variation between these clones was similar both in the terms of SRV and growth. ‘Matrix 49’ was also planted on R05, under dry conditions, and their differences between the growth were clearly visible (Table 6). Vusić et al. [55] described similarly weak growth in the case of ‘Hybride 275’, ‘Matrix 21’, and ‘Max 4’ in Croatia. ‘Bakan’ and ‘Skado’ have shown higher growth under the oceanic climate in Belgium than DN hybrids [56].

DD clones are research clones, but only planted on site R05. Research clone 1 was similar in the rate of survival and growth to clones with *P. maximowiczii* parentage, while there were better results with Research clone 2, which makes this clone a promising candidate under dry weather conditions and in poor soils. ‘AF7’ which is the only DNDT clone was planted only in one site as well but showed great SRV even though it was affected by browsing in the first year. *Populus alba* ‘Marte’ demonstrated high tolerance against water deficit in other studies [57], but in our case, this clone was highly preferred by game, which resulted in low SRV and a weak mean DBH and H (Table 6).

### Interaction Between Sites and Clones

**Fig. 3:**
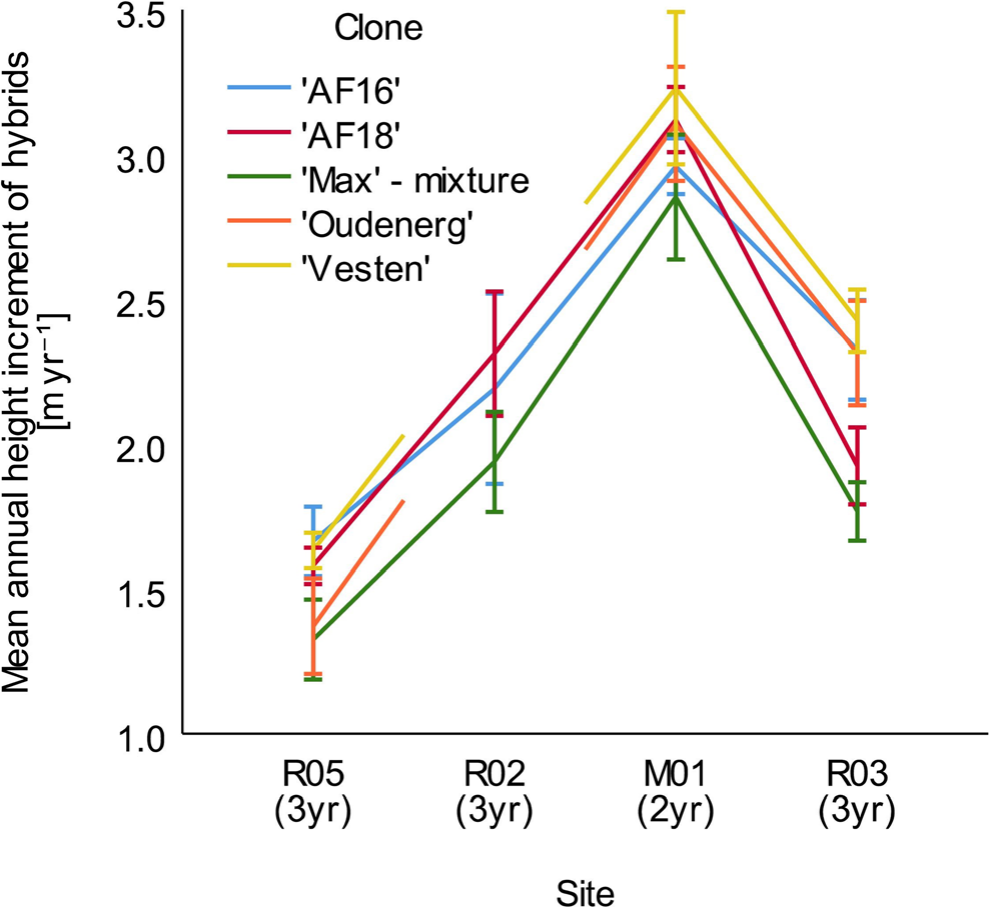
Mean annual height increment and standard errors of the five most common clones along the study sites, in the brackets number of survey years represented. R02, R03, and R05 were planted by rods while M01 was planted by cuttings

The most widely planted clones with sufficient survival in this study (‘AF16’, ‘AF18’, ‘Oudenberg’, ‘Vesten’, and ‘Max’ mixture) were compared, based on SRV and MAHI. The SRV of the different clones along the groundwater availability gradient—based on the groundwater level of sites (Table 2)—showed significant differences. Site (*F* (3, 49) = 51.518, *p* < .001) and clone factors (*F* (4, 49) = 2.895, *p* < .05) had significant effects on height increment as well. The site × clone interaction was not significant (*F* (10, 49) = .705, *p* = .715). This showed us that the growth of the clones on the different sites was similar. Post hoc tests revealed the similarity of R02 and R03. The clones had even showed different growths, but these differences were additive: different clones have a similar reaction to the availability of groundwater (Fig. 3). Post hoc tests showed that the means of ‘Max’ mixture and ‘Vesten’ are different, while the other clones were organized into homogeneous subsets both with ‘Max’ mixture or ‘Vesten’. The best growth was found at ‘Vesten’ along the gradient, but ‘AF16’ showed similar height increment on R05 site, but in the other cases its results were lower. ‘AF18’ grew better than ‘AF16’ at R02 and M01. ‘Oudenberg’ had similar results to ‘AF18’, but at R05 ‘Oudenberg’ grew at a lower rate and at R03 it grew in a higher rate. The ‘Max’ mixture showed the lowest values along the site gradient. Truax et al. [15] stated that inappropriate site selection cannot be compensated for by clone selection, which is confirmed by these findings.

## Conclusions

The differences in site quality and the different clones significantly affected SRV and growth. Firstly, we introduced the environmental factors which were the components of the site effect; secondly the average growth of the different clones on the sites based on the mean H and DBH; and lastly, the results of the site, clone, and interaction effects on selected hybrids.

The experimental test sites showed that the environmental factors were the most important regarding survival and growth of hybrid poplar at R05, R02, M01, and R03, especially the growing season precipitation and groundwater availability. These were the prime factors which highly affected the growth of different clones, while soil fertility—even at levels low for agricultural use—had a smaller role. The Site effect was large along the groundwater availability gradient. Clone selection proved to be a weaker and secondary factor. Site × Clone interaction was not significant, which showed that different clones reacted similarly to site factors. Some site factors were investigated, and others were not. Especially water availability can explain the differences of growth. Both on the levels of genomic groups and individual hybrids, growth gradually increased along the gradient, until the groundwater started to reduce the rooting zone as a result of the constant saturation. Then reductive processes were dominant, so growth was reduced. The best performing genomic group, the DN within ‘Vesten’ clone, was the highest yielding one and shortly behind it ‘AF18’, ‘Oudenberg’, and ‘AF16’.
